# A new petrophilous tiger beetle from the Trans-Pecos region of Texas and revised key to the genus *Amblycheila* (Coleoptera, Carabidae, Cicindelinae)

**DOI:** 10.3897/zookeys.893.47059

**Published:** 2019-12-02

**Authors:** Daniel P. Duran, Stephen J. Roman

**Affiliations:** 1 Department of Environmental Sciences, Rowan University, 201 Mullica Hill Rd., Glassboro, NJ 08028, USA Rowan University Glassboro United States of America; 2 178 Winecup Way, Austin, TX 78737, USA Unaffiliated Austin United States of America

**Keywords:** Caraboidea, Chihuahuan Desert, Nearctic, new species, Omini, petrophile, taxonomy

## Abstract

A new rock-dwelling (petrophilous) tiger beetle, *Amblycheila katzi* Duran & Roman, **sp. nov.** is described from calcareous canyons and steep hillsides in the Trans-Pecos region of western Texas. It is distinguished from all other *Amblycheila* based on multiple morphological characters, biogeography, and ecology. A revised key to the genus *Amblycheila* is provided.

## Introduction

The New World tiger beetle genus *Amblycheila* Say, 1829 includes seven currently recognized species (Wiesner 1992; Erwin and Pearson 2008; Pearson et al. 2015) exclusively within the Nearctic Realm, and is distributed from central and southwestern US to central Mexico. All members of the genus are nocturnal predators of invertebrates. They are found in desert or grassland ecosystems, with habitats including rolling hillsides in dry grasslands, rocky washes in deserts, and semi-open brush. Herein we describe *A. katzi* sp. nov., an inhabitant of steep rock canyons in the Trans-Pecos region of western Texas, part of the Chihuahuan Desert.

## Materials and methods

Specimens of an unidentified *Amblycheila* species from Val Verde County were collected by David Katz in 2010 and later by Daniel Sundberg in 2013; these were made available to the authors for study. Additional searches were performed by the authors and colleagues from 2014–2018, and in total 36 wild caught specimens were obtained. The second author collected live adults, 2^nd^ and 3^rd^ instar larvae from the type locality in 2015, and 59 additional adult beetles were reared through captive breeding. These specimens are indicated in the type series below by “*ex ovum*”.

Type material is deposited in the following institutional and private collections (acronyms used in the text are in parentheses): Smithsonian Institution, National Museum of Natural History (**NMNH**), Texas A&M University Insect Collection (**TAMUIC**), American Museum of Natural History Insect Collection (**AMNH**), Carnegie Museum of Natural History (**CMNH**), Florida State Collection of Arthropods (**FSCA**), Collection of David W. Brzoska (**DWBC**), Collection of Daniel P. Duran (**DPDC**), Collection of Stephen J. Roman (**SJRC**), Collection of David E. Katz (**DEKC**), Collection of Daniel Sundberg (**DSC**). Specimens of the new species were compared to material of all congeners, including *A. hoversoni* Gage, 1990, *A. picolominii* Reiche, 1840, *A. nyx* Sumlin, 1991, *A. halffteri* Mateu, 1974, *A. cylindriformis* (Say, 1823), *A. baroni* Rivers, 1890 and *A. schwarzi* Horn, 1904.

Body measurements are defined as follows. The total body length excludes the labrum and is measured as the distance from the anterior margin of the clypeus to the elytral apex, including the sutural spine, when present. The width of the pronotum is measured to include the lateral margins of the proepisterna. Pronotal width was measured at the widest point of the apex, as well at the narrowest point near the base.

## Taxonomy

### Amblycheila katzi

Taxon classificationAnimaliaColeopteraCarabidae

Roman & Duran sp. nov.

43136755-FA25-5D43-87FC-0163974AEAFF

http://zoobank.org/F2A07537-21DD-4371-A52F-2BAB80E8EF84

Figs 1 , 2 , 3

#### Material examined.

***Holotype***: 1 ♂, **USA**: Texas / Val Verde Co / 5mi E Langtry / 15-VI-2017 / leg. D. Katz (USNM). ***Paratypes***: 2 ♂♂, 2 ♀♀, same label data as holotype. 1♀, USA: Texas, 5mi W Langtry / 12-VII-2010 D. Katz (DEKC). 1 ♂, **USA**: Texas / Val Verde Co / Langtry / 18-VI-2013 / leg. D. Sundberg (DSC). 1 ♂, **USA**: Texas, / Val Verde Co / Langtry / 14-VI-2014 / leg. D. Sundberg (DSC). 1 ♂, **USA**: Texas, / Val Verde Co / Langtry / 28-XI-2014 / leg. D. Sundberg (DSC). 1 ♂, **USA**: Texas, / Val Verde Co / Langtry / 06-VII-2013 / leg. D. Sundberg (DSC). 1 ♂, **USA**: Texas, / Val Verde Co / Langtry / **-VII-2014 / leg. D. Sundberg (DSC). 1 ♂, **USA**: Texas, / Val Verde Co / 3.2mi W. Langtry / 21-VI-2014 / leg. D. Duran (DPDC). 5 ♂♂, 3 ♀♀, **USA**: Texas, / Val Verde Co / 4mi W Langtry / 18-VI-2015 / leg. S. Roman (SJRC). 1♀, **USA**: Texas, / Terrell Co / 13mi W Langtry / 18-VI-2015 / leg. S. Roman (SJRC). 3 ♂♂, 4 ♀♀, **USA**: Texas, / Val Verde Co / 3.5mi W Langtry / 01-VI-2017 / leg. S. Roman (SJRC). 2 ♂♂, **USA**: Texas, / Val Verde Co / 3.5mi W Langtry / 01-VI-2017 / leg. S. Roman (SJRC). 2 ♂♂, 2 ♀♀, **USA**: Texas, / Val Verde Co / 3.5mi W Langtry / 01-VII-2019 / leg. S. Roman (SJRC). 1 ♂, 2 ♀♀, **USA**: Texas, / Val Verde Co / 3.5mi W Langtry / 01-VII-2019 / leg. D. Katz (DKC). 1 ♂, **USA**: Texas, / Val Verde Co / 3.5mi W Langtry / 01-VII-2019 / leg. D. Katz (DKC). 4♂, 5 ♀♀, **USA**: Texas, / Val Verde Co / 3.5mi NW Langtry / 23-V-2016 / leg. D. Brzoska (DWBC). 1 ♂, **USA**: Texas, / Val Verde Co / 12.5mi NW Langtry / 23-V-2016 / leg. Brzoska (DWBC). 1 ♂ USA: Texas / Brewster Co / Heath Canyon / 22/IX/2019 / leg. J. Chong/S. Roman (SJRC). 6 ♂♂, 5 ♀♀, *ex ovum*, **USA**: Texas, / Terrell Co / 13mi W Langtry /

18-VI-15 / leg. S. Roman (SJRC). 24 ♂♂, 24 ♀♀, *ex ovum*, **USA**: Texas, / Val Verde Co / 13mi W Langtry / 18-VI-15 / leg. S. Roman (SJRC): 1 ♀ (USNM), 1 ♂, 1 ♀ (TAMUIC), 1 ♂, 1 ♀ (AMNH), 1 ♂, 1 ♀ (CMNH), 1 ♂, 1 ♀ (FSCA).

#### Diagnosis.

This species can be distinguished from all other *Amblycheila* by the combination of trapezoidal pronotum, almond-shaped elytra with sharply defined dorsal carinae, smooth and polished elytra with sparse irregular rows of setigerous punctures, the lack of a spine-line projection on the male hind trochanter, and small size for the genus (total body length 23–28 mm). No other geographically proximate congeners can be confused with this species. *Amblycheila schwarzi* is most similar in body shape and size, but its dorsal elytral texture is not smooth or polished, it possesses a greater number of shallow setigerous punctures that are arranged more regularly into rows between the dorsal carinae, and the two species are separated by 1300 km, with congeners occurring in the intervening areas.

#### Description.

Small-sized *Amblycheila*. Total body length 23.35–27.75 mm, mean ♀ 26.0 mm, mean ♂ 26.0 mm. ***Head*** (Fig. 2): slightly narrower than anterior margin of pronotum, black throughout, with dark reddish reflections under strong light, moderately polished, 2–8 supraorbital setae next to each eye. Eyes round, proportionately small, not protruding beyond the genae when viewed from above. Frons clearly delimited from clypeus by a visible suture, gradually blending into vertex. Frons surface mostly smooth, lacking striae, but broadly wrinkled with shallow depressions. Vertex smooth, flat, glabrous. Genae smooth, with 0–6 setae present on each side. Clypeus slightly convex, with 2 setae. Labrum black, convex, smooth with 6–12 setae (typically 8–10) set in deep punctae, length 3.0–3.8 mm, width 1.1–1.8 mm, bidentate. Mandibles black, medium-sized, each with 2–12 setae along outside margin. Maxillary palpi dark reddish brown to black, color consistent in each segment. Labial palpi as above. Antennae long, reaching apical third of elytron, slightly longer in male than female; scape dark testaceous to black, with 2 or more subapical setae, sometimes with additional non-apical setae; pedicel dark reddish brown to black, with 2 or more subapical setae; flagellum dark reddish brown, with ring of long apical setae, covered with fine pubescence throughout.

**Figure 1.**
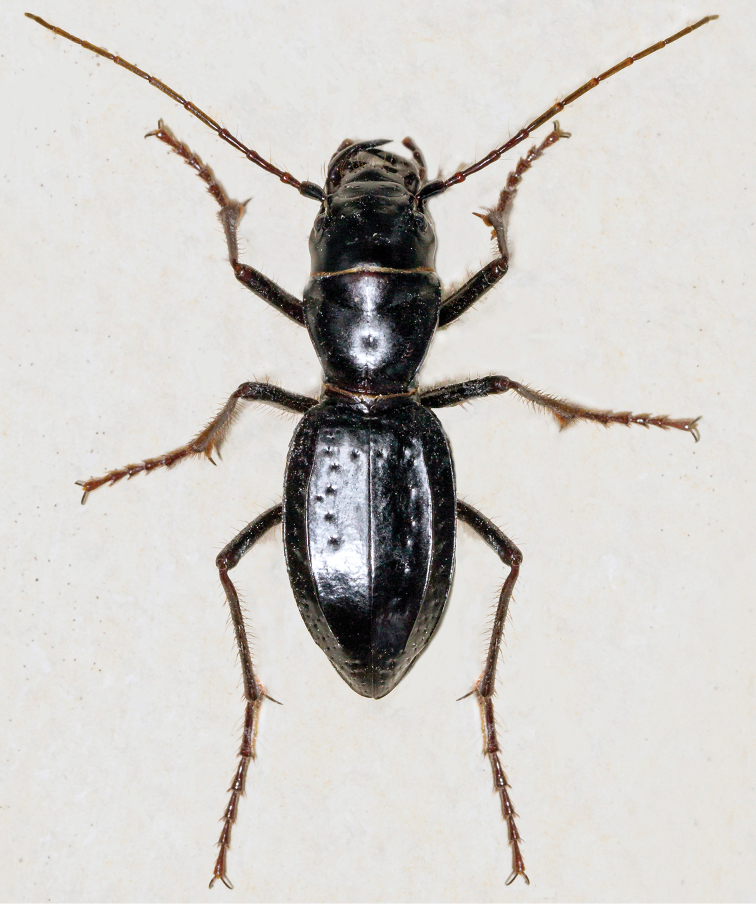
*Amblycheila katzi* Duran & Roman, sp. nov. dorsal habitus (female).

**Figure 2.**
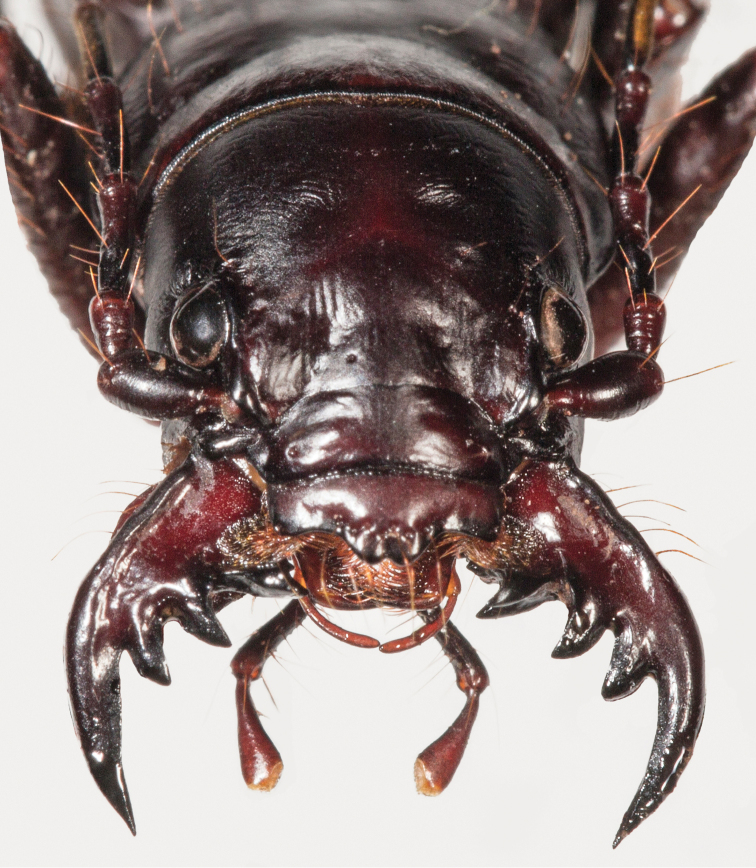
*Amblycheila katzi* Duran & Roman, sp. nov. frontal habitus (female).

***Thorax***: Pronotum 5.60–6.85 mm in width (mean = 6.30), black, slightly polished, nearly smooth, with fine shallow rugosity, especially near margins; trapezoidal in shape with nearly straight sides, widest near apex, 5.80–7.60 mm (mean = 6.95); base narrow, 4.00–5.30 (mean = 4.70), setae sparse (1–7 per side) and present along lateral third of dorsal surface; disc with thin weakly impressed median line and deeply impressed transverse line; notopleural sutures clearly defined, not visible from dorsal view; proepisternum black, dull, glabrous or with few sparse long setae mostly concentrated near the coxae; meso- and metasternum glabrous or with a few setigerous punctures, especially near coxae. Elytra amygdaloid, 13.60–16.40 mm length (mean = 15.15), shape similar in both sexes; sutural spine absent, micro-serrations absent; elytral dorsal surface smooth and slightly polished, with sparse and irregularly placed setigerous punctae present between dorsal carinae; additional small shallow setigerous punctures are present in lateral areas, especially between dorsal carinae and epipleura.

***Legs***: Pro-, meso- and metacoxae dark reddish brown to black, with sparse setae; trochanters dark reddish brown to black, pro- and mesotrochanter with a single long seta, sometimes with additional shorter setae; metatrochanter glabrous, apex blunt, lacking a produced apical tip; femora black, surface with multiple rows of erect brown setae; tibiae dark reddish brown to black, surface with erect brown setae similar to femora, with additional dense brown setae present at the apices near the tibial spines; tarsi dark reddish brown to black, bearing fine erect setae on lateral and ventral areas, tarsal claw long, simple.

***Abdomen***: Ventrites black, erect setae present and mostly restricted to a distinct row running parallel to the suture between ventrites, abdominal setae on ventrites 1–4 sparser in female that male; terminal abdominal ventrite with scattered setae near apical half in males, no setae present in females except for a dense fringe of setae emerging from the tip of the apex.

#### Etymology.

This new *Amblycheila* is named after David Katz, the discoverer of this remarkable petrophilous insect. We propose the common name of Trans-Pecos Giant Tiger Beetle.

#### Distribution and habitat.

**Figure 3.**
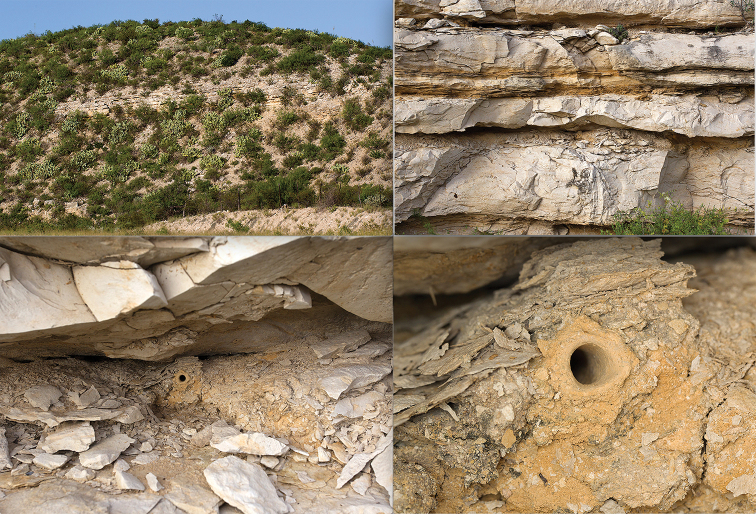
Habitat for *Amblycheila katzi* sp. nov. **A** steep-sided limestone road cut **B** rock ledges with grooves and cracks **C** rock ledge with calcareous soil and third instar *A. katzi* larval burrow **D** closeup of third instar *A. katzi* larval burrow opening.

*Amblycheila katzi* is currently known only from western Texas in the Trans-Pecos region of the Chihuahuan Desert. All known occurrences are from steep-sided canyons in Val Verde and Terrell Counties, where natural or man-made forces have exposed bedrock, primarily Cretaceous limestone (Freeman 1968) (Fig. 3A). This species appears to prefer vertical surfaces; the authors did not find any specimens on the ground, or in pitfall traps placed at the base of rock walls. All individuals observed by the authors were present 1–5m above the ground in rock crevices, grooves and ledges (Fig. 3B). Adult beetles were found in the same areas where *Latrodectus* spiders were abundant. The second author discovered 2^nd^ and 3^rd^ instar larvae in fine calcareous clays within grooves and crevices in vertical limestone walls (Fig. 3C, D).

##### Key to the genus *Amblycheila*

**Table:** 

1	One or two distinct carinae (pleats) running the length of each elytron (Fig. 4A)	**2**
–	Three distinct carinae (pleats) running the length of each elytron (Fig. 4B)	**3**
2	Elytral texture dull; indistinct shallow punctae present. Elytra tapering towards apex. Length 19–26 mm. Southeastern Arizona, northern Mexico	***A. baroni***
–	Elytral texture polished; indistinct shallow punctae present along with many small pinpoint punctures. Elytral apex bulbous. Length 26–31 mm. Northern Mexico, possibly southwestern Texas	***A. nyx***
3	Elytra densely covered with pinpoint punctures. Without rows of larger punctae between dorsal carinae (Fig. 5A)	**4**
–	Punctae between dorsal carinae loosely arranged into rows (Fig. 5B), not densely covering surface. May also possess smaller pinpoint punctures throughout	**5**
4	Elytra uniformly and densely punctate, surface texture dull to matte. Elytra dark red to dark brown. Length 29–40 mm. Great Plains region from South Dakota to Texas	***A. cylindriformis***
–	Elytral surface polished, bearing small shallow punctures with smooth edges. Elytra black. Length 29–33 mm. Central Mexico	***A. halffteri***
5	Pronotum rounded (Fig. 6A). Elytra reddish brown to black. Hind trochanter in male with elongated apex, spine-line. Length 25–37 mm	**6**
–	Pronotum trapezoidal, with nearly straight sides (Fig. 6B). Hind trochanter in male similar to pro- and mesotrochanter, blunt. Elytra black. Length 21–28 mm	**7**
6	Dorsum of each elytron with one well-defined longitudinal row of punctae, with a smaller number of additional irregular punctae; many small shallow pinpoint punctures present. Length 25–31 mm. Arizona to west Texas	***A. picolominii***
–	Dorsum of each elytron with deeply impressed punctae, arranged in multiple longitudinal rows or irregularly; shallow pinpoint punctures visible. Length 30–37 mm. South Texas	***A. hoversoni***
7	Elytral texture smooth and polished between sparse irregular setigerous punctae. Elytra nearly flat in lateral view, not bulging (Fig. 7A). Length 22–28 mm. Trans-Pecos region of West Texas	***A. katzi***
–	Elytral texture dull, not shining; with one or more longitudinal rows of shallow setigerous punctae. Elytra with bulge near apex, especially in male (Fig. 7B). Length 21–27 mm. Mojave Desert region of southeast California to southwest Utah and northwest Arizona	***A. schwarzi***

**Figure 4.**
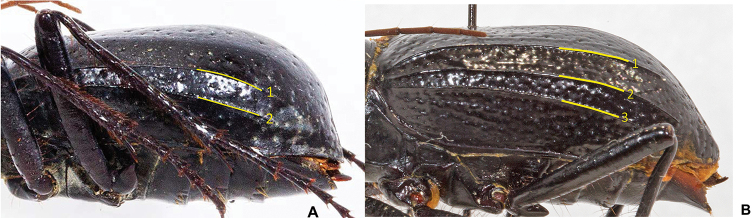
Carinae (pleats) present on *Amblycheila* elytra. **A** two distinct carinae (*A. nyx*) **B** three distinct carinae (*A. halffteri*).

**Figure 5.**
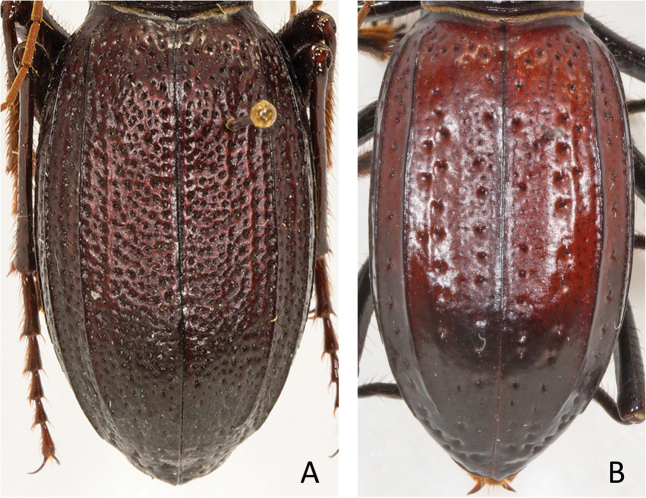
Elytral texture. **A** Dense pinpoint punctures present, without rows of larger punctae (*A. cylindriformis*) **B** with larger punctae loosely arranged into rows (*A. hoversoni*).

**Figure 6.**
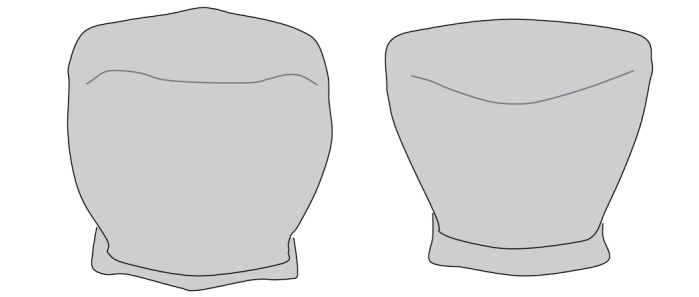
Pronotal shape **A** rounded **B** trapezoidal.

**Figure 7.**
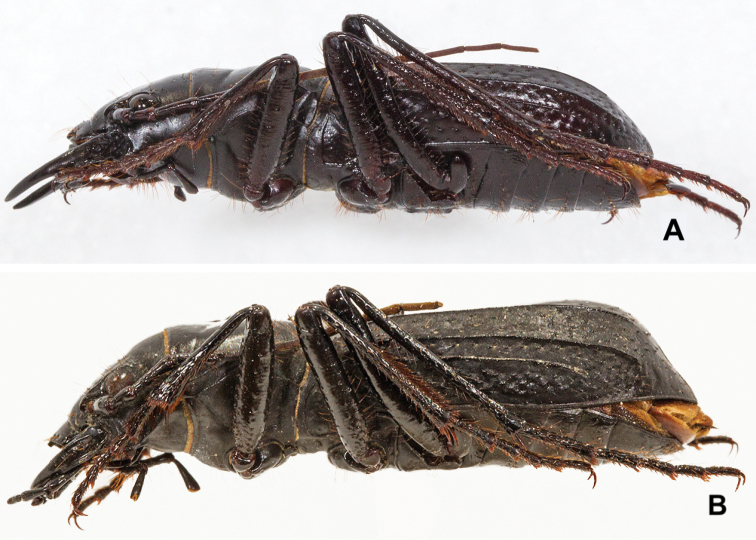
Lateral habitus. **A***Amblycheila katzi*, sp. nov. male with elytra gradually tapering towards apex **B***A. schwarzi*. male with elytral bulge and steep decline towards apex.

## Discussion

This species is likely more widespread than presently known. *Amblycheila katzi* can be at low densities, and is present in habitat that would not generally be checked by tiger beetle collectors. No other North American tiger beetle is apparently associated with vertical rock walls, and this unusual behavioral characteristic may further contribute to the lack of known specimens.

Given the fact that all four localities are very close to the US/Mexican border, it is a near certainty that this species occurs in northern Mexico as well. Only two of the eight *Amblycheila* species are known from Mexico, but this is likely due to collecting bias.

When constructing a dichotomous key to the genus *Amblycheila*, we reviewed the literature for potentially informative characters that could diagnose species (Horn 1910; Vaurie 1955; Mateu 1974; Gage 1990; Rumpp unpublished). Not all previously identified characters held up to scrutiny. We found that the placement and number of setae (chaetotaxy) were too variable within species to be of diagnostic value, a view shared by Vaurie (1955). Gage’s (1990) description of *A. hoversoni* indicates that it can be distinguished from all other *Amblycheila* based on the presence of a single row of punctae between the dorsal and lateral carinae. We examined long series of *A. hoversoni*, and this character was not always consistent, therefore we did not include it in our key.

## Supplementary Material

XML Treatment for Amblycheila katzi
